# Centrality and System Size Dependence among Freezeout Parameters and the Implications for EOS and QGP in High-Energy Collisions

**DOI:** 10.3390/e25121586

**Published:** 2023-11-26

**Authors:** Muhammad Waqas, Abd Haj Ismail, Haifa I. Alrebdi, Muhammad Ajaz

**Affiliations:** 1 School of Mathematics, Physics and Optoelectronic Engineering, Hubei University of Automotive Technology, Shiyan 442002, China; 2 College of Humanities and Sciences, Ajman University, Ajman P.O. Box 346, United Arab Emirates; 3 Department of Physics, College of Science, Prince Nourah Bint Abdulrahman Univeristy, P.O.Box 84428, Riyadh 11671, Saudi Arabia; 4 Department of Physics, Abdul Wali Khan University Mardan, Mardan 23200, Pakistan

**Keywords:** freezeout parameters, non-extensivity, qauntum chromodynamics, EOS, QGP

## Abstract

Utilizing the Modified Hagedorn function with embedded flow, we analyze the transverse momenta ($p_{T}$) and transverse mass ($m_{T}$) spectra of $\pi^{+}$ in Au–Au, Cu–Cu, and d–Au collisions at $\sqrt{s_{NN}}$ = 200 GeV across various centrality bins. Our study reveals the centrality and system size dependence of key freezeout parameters, including kinetic freezeout temperature $\left(T_{0}\right)$, transverse flow velocity $\left(\beta_{T}\right)$, entropy-related parameter (n), and kinetic freezeout volume (*V*). Specifically, $T_{0}$ and *n* increase from central to peripheral collisions, while $\beta_{T}$ and *V* show the opposite trend. These parameters also exhibit system size dependence; $T_{0}$ and $\beta_{T}$ are smaller in larger collision systems, whereas *V* is larger. Importantly, central collisions correspond to a stiffer Equation of State (EOS), characterized by larger $\beta_{T}$ and smaller $T_{0}$, while peripheral collisions indicate a softer EOS. These insights are crucial for understanding the properties of Quark–Gluon Plasma (QGP) and offer valuable constraints for Quantum Chromodynamics (QCD) models at high temperatures and densities.

## 1. Introduction

The collisions of heavy ions at relativistic energies in the laboratory allow the creation as well as the investigation of the hot and dense QCD matter [1,2,3]. The QCD phase diagram can be probed by tuning of collision energy, which enables the possibility of producing nuclear matter at various temperatures and baryon densities. The Relativistic Heavy Ion Collider (RHIC) [4,5] and Large Hadron Collider (LHC) [6,7,8] provide the opportunity to produce a medium that has the thermodynamic conditions of high temperatures and negligible baryon chemical potentials. This medium can be studied with high precision using the first-principle QCD calculations [9,10,11,12,13] within the Lattice QCD (lQCD) framework. The moderate temperature and finite net baryon densities in QCD can be created by lowering the beam energies. The application of lQCD to the study of such a matter is limited due to the so-called sign problem. However, there are current and future accelerator facilities, such as RHIC [14], Super Proton Synchrotron (SPS) [15,16], Nuclotron-based Ion Collider (NICA) [17], and the Facility for Anti-proton Ion Research (FAIR) [18,19], which have carried out or plan to conduct diverse experimental programs to explore this part of the QCD phase diagram. The sequence of events in relativistic heavy ion collisions involving the generation of hot and dense matter can be outlined as follows: a pre-equilibrium phase, the attainment of thermal (or chemical) equilibrium among partons, the potential formation of Quark–Gluon Plasma (QGP) or a mixed state of QGP and hadron gas, the emergence of a gas comprising hot interacting hadrons, and, ultimately, a freezeout state where the produced hadrons cease strong interactions. As the produced hadrons encapsulate information pertaining to the collision dynamics and the comprehensive spacetime evolution of the system from its initial to final stages, a precise assessment of transverse momentum ($p_{T}$) distributions and yields of identified hadrons in relation to collision geometry becomes crucial for comprehending the dynamics and properties of the generated matter.

The freezeout conditions of the fireball have great importance and have been one of the compelling topics in the study of heavy ion collisions at various energies and in different centrality intervals. From the analysis of two-particle correlations [20,21] and hadron yields, the freezeout is claimed to occur in two stages: (1) chemical freezeout, where the particle ratio stabilizes as the inelastic scattering stops; and (2) kinetic freezeout, where the momentum distribution of the particles is frozen.

The kinetic freezeout stage is very important in the evolution of heavy ion collisions because it provides information about the properties of nuclear matter and the underlying dynamics of the strong interactions. Different hydrodynamic models [22,23,24,25,26] can be used to investigate the hot and dense matter in terms of various parameters to be extracted. In the present work, the ($p_{T}$) spectra of pions in Au–Au, Cu–Cu, and d–Au interactions at 200 GeV in several centrality intervals are analyzed by the Modified Hagedorn function with the embedded flow to extract $T_{0}$, $\beta_{T}$, *n*, and *V*. All these parameters are discussed in our previous works in detail [27,28,29,30]. The $T_{0}$ is the temperature at which the QGP is already transformed into a gas of hadrons and the interactions between the particles cease. The $\beta_{T}$ is the collective motion of the particles in the transverse direction, perpendicular to the beam axis, due to the pressure gradients within the QGP. It should be noted that we took pions because they are the most abundant particles that are produced in collisions.

The subsequent sections of the paper follow this structure: Section 2 outlines the methodology and formal framework, Section 3 delves into the discussion of results, and Section 4 provides the concluding remarks.

## 2. The Method and Formalism

The $p_{T}$ parameters of the final state particles have great importance in high-energy physics and are distributed among several components. These components include the soft, hard, very soft, and very hard components, which are discussed in detail in our previous work [31]. Let us bind our discussion to the soft and hard components. Several functions and distributions may be used to describe the $p_{T}$ spectra. Some distributions may describe soft components, while some of them may be used to describe both the soft and hard components. The $p_{T}$ range of 0–2 or 2.5 GeV/c can be referred to as the soft component, while the range above that is considered the hard component.

Various versions of the Tsallis distribution function, rooted in non-extensive Tsallis statistics, have become widely used models for describing the $p_{T}$ distributions of hadrons in high-energy collisions [32,33,34,35]. Unlike others, the Tsallis function offers a distinct advantage: it is directly linked to thermodynamics through entropy [35]. The Tsallis function includes a crucial parameter, the non-extensivity index *q*, which indicates how much the particle $p_{T}$ distribution deviates from the Boltzmann–Gibbs exponential distribution. Additionally, the parameter *q* serves as a measure of the system’s departure from equilibrium or thermal equilibrium [36]. The significance of *q* and its profound physical implications, directly related to thermodynamics, have been reaffirmed in recent research by Tsallis [33].

The Tsallis function at mid-rapidity in its most basic form is provided as [37,38]

(1) $$f\left(p_{T}\right)=C{\left(1+\left(q-1\right)\frac{m_{T}}{T}\right)}^{-1/(q-1)},$$

*C* denotes the normalized constant, while *T* represents the effective temperature. This temperature, encompassing the flow effect, is defined as $T=\sqrt{\frac{T_{0}\left(1+\beta_{T}\right)}{\left(1-\beta_{T}\right)}}$. As cited in [38,39,40], the Tsallis distribution, expressed in the following form, aligns with thermodynamic principles:

(2) $$f\left(p_{T}\right)=C{\left(1+\left(q-1\right)\frac{m_{T}}{T}\right)}^{-q/(q-1)},$$

The $\beta_{T}$ is incorporated into a QCD-inspired (power law) Hagedorn function using a straightforward Lorentz transformation [41,42]. This approach effectively replicated the observed extended ranges of momentum spectra for final particles in both heavy-ion and pp collisions at high energies.

For the description of the hard component of the $p_{T}$ spectra, one may use the Hagedorn function [43], which is described by the inverse power law [44,45,46]

(3) $$\frac{1}{N}\frac{d^{2}N}{2\pi p_{T}dp_{T}dy}=C{\left(1+\frac{m_{T}}{p_{0}}\right)}^{-n},$$

and

(4) $$m_{T}=\sqrt{{\left(m_{0}\right)}^{2}+{\left(p_{T}\right)}^{2}},$$

In the given context, N denotes the number of particles, and $p_{T}$ ($m_{T}$) represents the transverse momentum (mass) of these particles. The parameters $p_{0}$ and *n* are variables allowed to vary freely during the fitting process, with the latter expressed as $n={\left(q-1\right)}^{-1}$. The value $m_{0}$ corresponds to the rest mass of the pion, which is 0.139 GeV/$c^{2}$ [47].

Equations (1) and (3) are mathematically identical when one sets $p_{0}$ = $nT_{0}$ and *n* = ${\left(q-1\right)}^{-1}$. So, Equation (3) becomes

(5) $$\frac{1}{N}\frac{d^{2}N}{2\pi p_{T}dp_{T}dy}=C{\left(1+\frac{m_{T}}{nT_{0}}\right)}^{-n}.$$

In the current work, the simplest transformation is used to incorporate the collective transverse (radial) flow into Equation (5) $m_{T}$⟶$<\gamma_{t}>$${\left(m_{T}-p_{T}/\beta_{T}\right)}^{-n}$, such that Equation (5) becomes

(6) $$f\left(p_{T}\right)=C{\left(1+\frac{<\gamma_{T}>\left(m_{T}-p_{T}<\beta_{T}>\right)}{nT_{0}}\right)}^{-n},$$

This is the Hagedorn function with embedded flow, where *C* = $gV/{\left(2\pi \right)}^{2}$ is the normalization constant and *V* is the kinetic freezeout volume. $T_{0}$ and $\beta_{T}$ represent the kinetic freezeout temperature and transverse flow velocity, respectively. *n* is a parameter that is related to non-extensivity, and $\gamma_{t}=1/\sqrt{1-<\beta_{T}{>}^{2}}$. One can further read about the Hagedorn model with the embedded flow in Refs. [41,48]. Before proceeding to the next section, we would like to clarify that, if the hard component is included, we can apply the superposition principle to combine Equations (3) and (6), as indicated by references [27,31].

## 3. Results and Discussion

**Figure 1:**
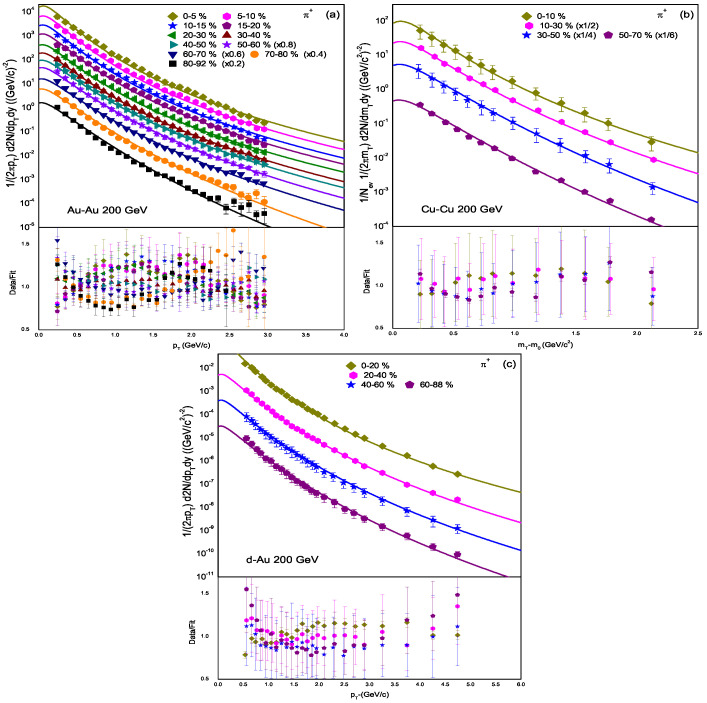
$p_{T}$ spectra of $\pi^{+}$ produced in (**a**) Au–Au, (**b**) Cu–Cu, and (**c**) d–Au collisions in various centrality intervals at $\sqrt{s_{NN}}$ = 200 GeV. The experimental data from the PHENIX and BRAHMS collaborations are taken from [49,50,51], while the solid lines represent the fit results of the model. The lower segment in each panel provides the data/fit.

The $p_{T}$ ($m_{T}$) spectra of $\pi^{+}$ in Au–Au, Cu–Cu, and d–Au collisions at $\sqrt{s_{NN}}$ = 200 GeV are presented in Figure 1. We have analyzed $p_{T}$ spectra in various centrality bins. The data are taken from [49,50,51], denoted by different symbols for different centrality intervals. One can see that the model provides a good fit to the experimental data. The values of the extracted parameters and $\chi^{2}$ are presented in Table 1. The data/fit in the lower segment of each panel, and the values of $\chi^{2}$ show the quality of the fit. The normalization constant *C* is integrated into the equations to normalize them to unity, while $N_{0}$ is used to compare the experimental data with the model fit and is considered as the multiplicity parameter.

**Figure 2:**
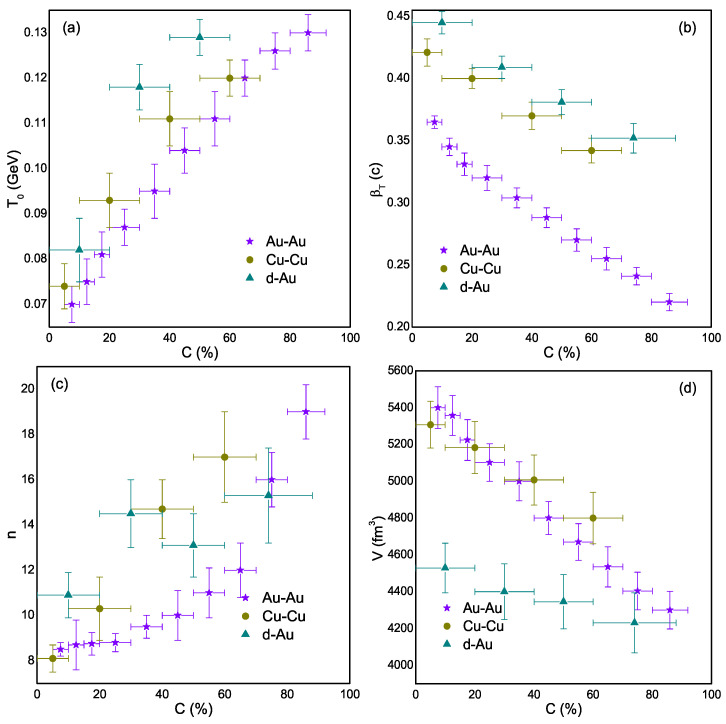
$T_{0}$, $\beta_{T}$, *n*, and *V* are shown in centrality classes in panels (**a**), (**b**), (**c**), and (**d**), respectively. Different symbols with different colors in all four panels of Figure 2 demonstrate different collision systems. The change in these symbols towards the right shows their dependence on centrality.

The extracted parameters, $T_{0}$, $\beta_{T}$, *n*, and *V*, as a function of centrality and system size, are shown in Figure 2. Figure 2a shows that $T_{0}$ increases toward peripheral collisions, indicating that the fireball lifetime decreases towards the peripheral collisions. On the other hand, $\beta_{T}$ decreases as we move to non-central collisions as the pressure gradient decreases toward peripheral collisions. We know that $T_{0}$ in heavy ion collisions is sensitive to the thermal and dynamical properties of the created system and $\beta_{T}$ characterizes the collective motion of the particles in the transverse direction. The fluctuations in these quantities are determined by the interplay between the preliminary conditions, the expansion dynamics, and the freezeout process. Basically, in peripheral collisions, the weak pressure gradients result in a more gradual cooling of the system and, hence, lower $\beta_{T}$, and the particle density decreases more slowly, which results in larger $T_{0}$ in peripheral collisions compared to central collisions. Therefore, larger $T_{0}$ corresponds to smaller $\beta_{T}$ in peripheral collisions, indicating a short-lived fireball with a steady expansion of the system. Our results agree with the STAR results at 200 GeV [52], but the specific parameter values differ. The parameters obtained by BRAHMS [50] are relatively larger than ours. The variation in parameter values is attributed to different models. These findings are also consistent with those obtained from the blast wave model [52], accurately reflecting the physical reality of the collisions. Our model includes the non-extensive parameter, which offers a more suitable description of particle spectra in extreme conditions and accounts for deviations from equilibrium in non-extensive systems. The disparity between the $T_{0}$ values in our work and the chemical freezeout temperature extracted from the statistical and thermal models [49] is substantial. This difference may be due to the complex dynamics and non-equilibrium effects in high-energy systems, such as 200 GeV, which encompass processes like hadronization and hadronic rescattering. Precisely measuring chemical and kinetic freezeout temperatures in experiments is challenging, and the discrepancy in these temperatures underscores the difficulty in extracting these values from experimental data.

We also see (Figure 2a) that $T_{0}$ depends on the colliding system’s size. For a larger system, the $T_{0}$ is smaller. Similarly, in Figure 2b, $\beta_{T}$ has the same behavior as the system size. Large colliding nuclei can provide a larger volume of the system, which results in a longer expansion time and a lower energy density at the time of kinetic freezeout. This leads the particles to have less time to interact and thermalize with each other, leading to a lower $T_{0}$. On the other hand, we know that $\beta_{T}$ refers to the collective motion of the particles in the transverse direction, perpendicular to the beam axis. This velocity can be generated by the pressure gradients created by the initial collision and subsequent expansion of the system. Therefore, a larger $\beta_{T}$ can also correspond to a smaller system size as the particles will be more spread out in the transverse direction due to their collective motion. The smaller $\beta_{T}$ for large systems in the current work can be explained in terms of, in larger collision systems, there is typically a higher initial energy density, which can lead to a longer duration of the early dense stage of the collision. Additionally, because of the longer interaction time and larger system size, the expansion can be more gradual and less violent. As a result, the transverse flow velocity may increase more slowly.

Figure 2c provides the result of the dependence of *n* on centrality. Basically, *n* = ${\left(q-1\right)}^{-1}$, and *q* is the non-extensive parameter [36,53]. The parameter *q* is used to explain the deviation from thermal equilibrium and can be used for quantification of the fluctuations in temperature around the equilibrated value of temperature. The parameter *q* and temperature can be interconnected as

(7) $$q-1=\frac{Var(T_{0})}{<T_{0}>}$$

Larger (small) *q* refers to a large (small) deviation in the system from thermal equilibrium, where larger *q* corresponds to smaller *n*. In the present work, the central collisions are far from thermal equilibrium because the value of *n* is smaller in central collisions, and it increases toward peripheral collisions, which means that the peripheral collisions are closer to equilibrium. The above statement seems unusual but it is not. It is possible that the peripheral collisions may have a closer approach to equilibrium than the central collision systems, which can be explained in terms of higher energy densities and more violent interactions being experienced by the system in central collisions, where there is a greater overlap between the colliding nuclei. This may cause the system to expand and cool quickly, which could shorten the amount of time it takes for the particles to reach thermal equilibrium. Central collisions may therefore show non-equilibrium features. Peripheral collisions, on the other hand, involve lower energy densities and less overlap. The system can evolve more slowly in peripheral collisions due to the longer interaction times, even though the overall energy deposited is lower. The system may become more “equilibrated” in terms of conventional thermodynamic properties as a result of this prolonged evolution, which may give the particles more chances to achieve a state of thermal equilibrium.

The dependence of (V) is shown in Figure 2d. One can see that *V* depends on both the system size and collision centrality. This occurs because central collisions are associated with larger initial bulk systems at higher energies. This, in turn, results in longer evolution times and the formation of larger partonic systems. Naturally, a larger partonic system corresponds to a larger *V*. Meanwhile, the scenario is the opposite regarding the periphery, where *V* becomes smaller. *V* is also dependent on the system size. The larger the system, the larger the *V*. The fact behind this is that a large number of particles are produced in larger systems; as a result, larger volume is required to accommodate these particles at the time of kinetic freezeout.

**Figure 3:**
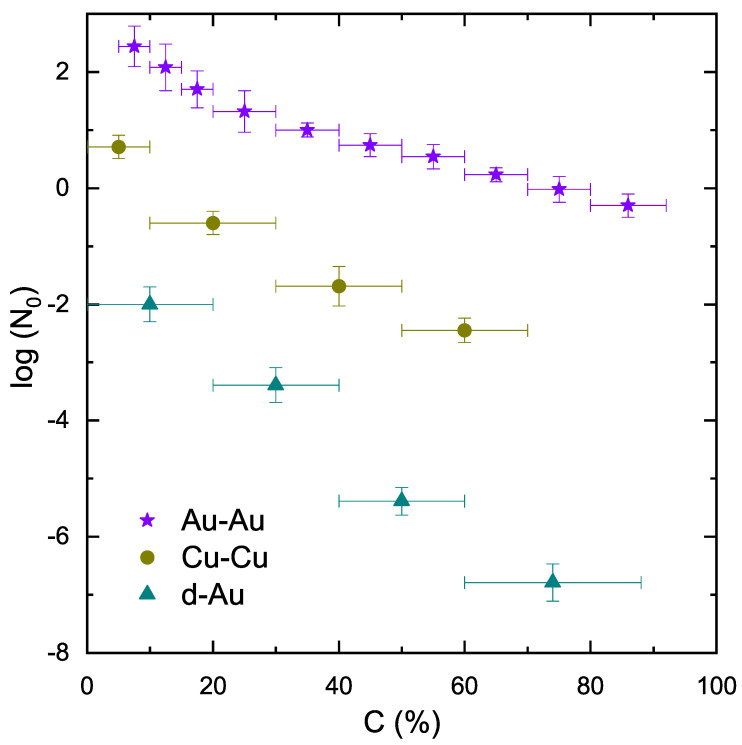
The variation in $N_{0}$ with centrality and size of the interacting system.

Figure 3 shows the multiplicity parameter ($N_{0}$) as a function of centrality and the size of the collision system. Central collisions correspond to large multiplicity because the overlapping region contains huge energy during the collision. At the time of ion collision, a high-temperature and high-density medium of quarks and gluons known as the QGP is produced. This plasma quickly expands and cools, eventually breaking up into a large number of particles. In other words, the multiplicity of particles produced in the central collisions is related to the energy density of the QGP. High energy density means that there are more particles per unit volume, leading to a larger number of particles. This is why central collisions, which have the highest energy densities, are more likely to produce a large number of particles. When the centrality decreases, the energy densities in the system also decrease, which results in smaller multiplicity.

Before advancing to the conclusion, we would like to emphasize that the present work is very important because, in heavy ion collisions, the $T_{0}$ and $\beta_{T}$ are two important observables that are related to the EOS of the QGP. The EOS describes the relationship between the thermodynamic variables of the QGP, such as temperature, pressure, and energy density.

The relationship between the $T_{0}$ and the $\beta_{T}$ can be used to constrain the EOS of the QGP. In this work, the higher values of $\beta_{T}$ and smaller values of $T_{0}$ in the highest centrality correspond to a stiffer EOS, showing large pressure. The stiffer EOS is due to a large pressure gradient and lower $T_{0}$, which will lead to a faster expansion of the QGP and a larger pressure gradient, resulting in greater collective motion of the particles. Conversely, peripheral collisions correspond to a softer EOS, which shows a slower expansion. The softer EOS corresponds to lower pressure for a given energy density, which will result in a smaller $\beta_{T}$ and a higher $T_{0}$. This is because a softer EOS will lead to a slower expansion of the QGP and a smaller pressure gradient, resulting in lesser collective motion of the particles. A stiffer EOS indicates a stronger interaction between the quarks and gluons in QGP, whereas a softer EOS indicates a weaker interaction. This information is important for understanding the properties of the QGP and for constraining theoretical models of QCD at high temperatures and densities.

## 4. Conclusions

The transverse momentum (mass) spectra of $\pi^{+}$ at $\sqrt{s_{NN}}$ = 200 GeV in different centrality bins of Au–Au, Cu–Cu, and d–Au collisions are analyzed, and the freezeout parameters are extracted. The extracted parameters are the $T_{0}$, $\beta_{T}$, kinetic freezeout, and the non-extensive parameter.

We presented the dependence of the extracted parameters on centrality as well as on the size of the interacting system. The $T_{0}$ shows a declining trend from peripheral to central collisions, which shows a short-lived fireball in central collisions. On the other hand, the $\beta_{T}$ shows an opposite trend from peripheral to central to peripheral collisions, which suggests a large pressure gradient in a central collision that results in a quicker expansion of the system. The $T_{0}$ and $\beta_{T}$ have a negative correlation. The larger the $T_{0}$, the smaller the $\beta_{T}$. Furthermore, the *V* follows the trend of the $\beta_{T}$, which indicates that a greater number of participant nucleons take part in central collisions. The parameter *n* follows the trend of the $T_{0}$, showing that the peripheral collisions come to an equilibrium state easily. The above parameters also depend on the size of the colliding system. Large colliding systems have smaller $T_{0}$ and $\beta_{T}$, and larger *V*.

## Figures and Tables

**Table 1:** Collision, centrality, the extracted parameters ($T_{0}$, $\beta_{T}$, and *n*), fitting constant ($N_{0}$), $\chi^{2}$, and degrees of freedom (dof) corresponding to the graphs in Figure 1.

Collision	Centrality	$T_{0}$ (GeV)	$\beta_{T}$	*n*	$V({\mathrm{fm}}^{3})$	$N_{0}$	$\chi^{2}$/dof
Au–Au	0–5%	0.065±0.004	0.380±0.008	8.4±0.5	5568±131	680±37	31/25
	5–10%	0.070±0.004	0.365±0.005	8.5±0.3	5400±113	280±28	28/25
	10–15%	0.075±0.005	0.345±0.007	8.7±1.1	5357±108	120±19	76/25
	15–20%	0.081±0.005	0.331±0.009	8.75±0.5	5224±111	55±9.2	15.8/25
	20–30%	0.087±0.004	0.320±0.010	8.8±0.4	5102±102	21±4	11/25
	30–40%	0.095±0.006	0.304±0.008	9.5±0.5	5000±106	10±0.6	3.3/21
	40–50%	0.104±0.005	0.288±0.008	10±1.1	4800±90	5.5±0.4	13.4/25
	50–60%	0.111±0.006	0.270±0.009	11±1.1	4670±100	3.5±0.3	5.8/25
	60–70%	0.120±0.004	0.255±0.009	12±1.2	4535±109	1.7±0.22	2/25
	70–80%	0.126±0.004	0.241±0.007	16±1.2	4404±102	0.95±0.08	159/25
	80–92%	0.130±0.004	0.220±0.007	19±1.2	4300±102	0.5±0.04	57/25
Cu–Cu	0–10%	0.074±0.005	0.421±0.011	8.1±0.6	5307±127	5.2±0.3	1/10
	10–30%	0.093±0.006	0.400±0.008	10.3±1.4	5183±141	0.25±0.04	0.4/10
	30–50%	0.111±0.006	0.370±0.011	14.7±1.3	5007±136	0.02±0.004	1/10
	50–70%	0.120±0.004	0.342±0.010	17±2	4800±139	0.0035±0.0005	1.3/10
d–Au	0–20%	0.082±0.007	0.445±0.009	10.9±1	4529±135	0.01±0.003	4/21
	20–40%	0.118±0.005	0.409±0.009	14.5±1.5	4400±152	$5\times 10^{-4}\pm 4\times 10^{-5}$	7/23
	40–60%	0.129±0.004	0.381±0.010	13.1±1.4	4346±147	$4\times 10^{-6}\pm 6\times 10^{-7}$	3/21
	60–88%	0.142±0.006	0.352±0.012	15.3±2.1	4231±163	$1.6\times 10^{-7}\pm 5\times 10^{-8}$	7.2/21

## Data Availability

All data generated or analyzed during this study are included and cited in this article.
